# Quantifying the contrast of the human locus coeruleus *in vivo* at 7 Tesla MRI

**DOI:** 10.1371/journal.pone.0209842

**Published:** 2019-02-06

**Authors:** Klodiana-Daphne Tona, Matthias J. P. van Osch, Sander Nieuwenhuis, Max C. Keuken

**Affiliations:** 1 Cognitive Psychology Unit, Institute of Psychology and Leiden Institute for Brain and Cognition, Leiden University, Leiden, The Netherlands; 2 Clinical Psychology Unit, Institute of Psychology, Leiden University, Leiden, The Netherlands; 3 Department of Radiology, Leiden University Medical Center and Leiden Institute for Brain and Cognition, Leiden University, Leiden, The Netherlands; 4 Integrative Model-Based Cognitive Neuroscience Research Unit, University of Amsterdam, Amsterdam, The Netherlands; Brigham and Women's Faulkner Hospital, UNITED STATES

## Abstract

The locus coeruleus is a small brainstem nucleus which contains neuromelanin cells and is involved in a number of cognitive functions such as attention, arousal and stress, as well as several neurological and psychiatric disorders. Locus coeruleus imaging *in vivo* is generally performed using a T_1_-weighted turbo spin echo MRI sequence at 3 Tesla (T). However, imaging at high magnetic field strength can increase the signal-to-noise ratio and offers the possibility of imaging at higher spatial resolution. Therefore, in the present study we explored the possibility of visualizing the locus coeruleus at 7T. To this end, twelve healthy volunteers participated in three scanning sessions: two with 3T MRI and one with 7T MRI. The volumes of the first 3T session were used to segment the locus coeruleus, whereas the volumes of the second 3T and the 7T session were used to quantify the contrast of the locus coeruleus with several reference regions across eight different structural sequences. The results indicate that several of the 7T sequences provide detectable contrast between the locus coeruleus and surrounding tissue. Of the tested sequences, a T_1_-weighted sequence with spectral presaturation inversion recovery (SPIR) seems the most promising method for visualizing the locus coeruleus at ultra-high field MRI. While there is insufficient evidence to prefer the 7T SPIR sequence over the 3T TSE sequence, the isotropic voxels at 7T are an important advantage when visualizing small structures such as the locus coeruleus.

## Introduction

The locus coeruleus (LC) is a brainstem nucleus that is involved in important cognitive functions such as attention, arousal and stress [1]. Also LC atrophy has been connected to disorders such as Alzheimer’s and Parkinson’s disease [2, 3]. The important role that the LC plays in cognition and its use as a biomarker for assessment of neurodegenerative disorders [4–7] necessitate accurate visualization of the LC.

Recently, a T_1_-weighted FLASH sequence has been used to image the LC at 3T [8]. However, to date the most frequently used scan at 3T scanners is a T_1_-weighted TSE scan sequence (e.g. [8, 9, 10–12]). Such a TSE sequence is thought to be sensitive to neuromelanin, a pigment that is produced in catecholaminergic neurons and that exists in large quantities in the LC [13]. Indeed, the TSE scan shows enhanced contrast between neuromenalin-rich structures such as the LC and surrounding brain tissue [6, 14]. The LC contrast is thought to be attributed to a number of tissue properties which selectively influence the MRI contrast mechanisms. The presence of neuromelanin alone can result in paramagnetic T_1_-shortening effects [15]. Paramagnetic ions such as those of iron and copper found in the LC are expected to contribute to T2 and T2* contrast [16–18].

While the TSE sequence has moderate test-retest reliability for visualizing the LC at 3T [19], the voxel dimensions generally acquired are highly anisotropic, which hinders the accurate visualization of the LC [20]. We hypothesized that imaging at higher magnetic field strength might provide a solution to the challenges involved in LC imaging. Higher magnetic field strength increases signal-to-noise ratio and allows imaging at a higher spatial resolution [21–23]. This, in turn, results in smaller partial volume effects, which in itself can help to improve contrast and thereby detectability [24, 25].

However, it is well-known that MRI properties of tissue are field-strength-dependent [23] and that the TSE sequence is specific absorption rate (SAR) intensive [26]. Therefore, it is uncertain whether sequences that have been proven successful at 3T, such as the TSE scan, will also provide the best contrast for visualization of the LC at higher field strengths. To address this issue, we studied the contrast of the LC for a number of 7T MRI sequences that have proven to provide detailed anatomical information in the brainstem at 7T [27] or which have been suggested for imaging neuromelanin or catecholamine-related structures such as the LC and the nigrosome substructures of the substantia nigra [28–31]. See also methods for more details on the scan sequences and choice rationale.

Our aim was to explore whether there are 7T scan sequences that can achieve adequate contrast for imaging the LC. For reference, we also included the standard TSE sequence used at 3T. The results indicate that several of the 7T sequences provide detectable contrast between the locus coeruleus and surrounding tissue. Of the tested sequences, a T_1_-weighted sequence with spectral presaturation inversion recovery (SPIR) seems the most promising method for visualizing the locus coeruleus at ultra-high field MRI.

## Methods

### Participants

Twelve healthy volunteers (mean age 23 years old; SD: 1.7) participated in three scanning sessions: two at 3T MRI and one at 7T MRI (the two 3T scan sessions were initially acquired to assess reproducibility of the TSE scan at 3T MRI, results published in [19]). All participants were healthy, right-handed and without a history of neurological or psychiatric problems as assessed by self-report questionnaires. The study was approved by the medical ethics committee of the Leiden University Medical Center. All participants provided written informed consent prior to their inclusion in the study (in accordance with the Declaration of Helsinki), and received monetary compensation for their participation.

### Choice of MRI scan sequences

The following scan sequences for 7T MRI were included in this study. Details about each scanning protocol are provided in the section “MRI acquisition parameters”.

- TSE sequence (TSE 7T): this sequence closely resembles the neuromelanin-sensitive scan as commonly employed at 3T [6, 19]. Minor adaptations were necessary to allow the usage of this scan at 7T, mainly due to specific absorption rate limits.
- High-resolution TSE sequence (HR-TSE 7T): this scan is similar to the previous TSE scan but at a higher spatial resolution, with a higher in-plane resolution, as well as a continuous coverage (no slice gap) in the z-direction, which was achieved via acquisition in four packages of interleaving slices.
- High-resolution T_2_*-weighted sequence: this scan is commonly used at 7T for detection of iron deposition, presence of deoxyhemoglobin, or other sources of magnetic susceptibility variations (HR-T2*; [28, 32]). For this scan, both the magnitude and phase of the MRI signal were recorded, which allows the reconstruction of three image types:
  - The magnitude image (HR-T_2_* magnitude).
  - The unwrapped phase image (HR-T_2_* phase unwrapped) for which a high-pass filter was applied to exclude low spatial-frequency variations of the phase due to macroscopic magnetic field differences.
  - The susceptibility-weighted images (HR-T_2_* SWI), based on the multiplication of the magnitude image with a filtered version of the unwrapped phase image, as originally proposed to enhance venous structures [29].
- T_1_-weighted scan with spectral presaturation with inversion recovery sequence (SPIR): this scan has previously been used in Parkinson’s disease patients at 7T to directly visualize nigrosome substructures of the substantia nigra by utilizing the presence of iron and neuromelanin in this structure [31]. SPIR is a hybrid technique which nulls the fat magnetization by means of a fat-selective radio frequency pulse and spoiler gradient. Due to the fact that the SPIR pulse is off-resonance with respect to the water peak, this pulse exhibits some magnetization transfer (MT) effects on the contrast [31].
- A whole-brain 3D T_1_-weighted (MPRAGE) sequence. This is a widely-used pulse sequence for anatomical imaging in MRI with contrast originating from T_1_ relaxation time differences.

Furthermore, at 3T we acquired the T_1_-weighted TSE structural scan that has been used to accentuate neuromelanin rich structures [6, 14]. The TSE scan from the first 3T scan session was used to manually segment the LC according to the segmentation protocol described in Tona et al., [19]. This LC mask was used to localize the LC in the 7T images and in the TSE image of the second 3T scan session. The TSE scan from session 2 was used to extract the 3T TSE signal and provided the reference value for the contrast extraction at 7T. Because the 3T TSE from session 2 is separate from the scans which were used to create the LC masks (first 3T TSE scan session), a comparison between the contrasts at 3T and 7T can be made while trying to avoid biases towards the sequence of the scan on which the mask was drawn.

### MRI acquisition parameters

**3 Tesla MRI** Two scanning sessions took place at a 3T-TX Philips scanner equipped with a 32-channel head coil. During both scan sessions, the participants underwent a whole-brain 3D T_1_-weightedsequence (3T whole-brain T1; field of view (FOV): 224 x 177.33 x 168 mm; 140 slices; resolution 0.87 x 0.87 x 1.2 mm; repetition time (TR): 9.7ms; echo time (TE): 4.5 ms; flip angle 8^o^; acquisition matrix: 192 x 152; time of acquisition (TA): 4 min 9 s) and a brainstem-zoomed T_1_-weighted TSE structural scan [6]. The TSE scan sequence was used to detect the LC and had similar sequence parameters as the ones reported in prior literature [12] (FOV: 180 x 180 x 22.95 mm; 14 slices; acquisition resolution 0.70 x 0.80 x 1.5 mm, reconstruction resolution (through the use of zero padding) 0.35 x 0.35 x 1.5 mm, gap of 10%; TSE factor: 3; repetition time (TR): 500 ms; echo time (TE): 10 ms; flip angle 90^o^; acquisition matrix: 256 x 204; time of acquisition (TA): 7 min). For more details about the 3T methods see Tona *et al*. (2017).

**7 Tesla MRI** The 7T MRI session was performed on a whole-body Achieva Philips scanner equipped with a 32-channel head coil. During this scan session, the participants underwent a whole-brain 3D T_1_-weighted scan (FOV: 246 x 246 x 174 mm; 249 slices; 0.69 x 0.69 x 0.69 mm; TR: 4.5 ms; TE: 2.0 ms; flip angle 7^o^; acquisition matrix: 352 x 353; TI = 1600 ms; sagittal orientation; TA: 8 min 26 s).

We acquired a TSE scan with parameters as similar as possible to the 3T scan (TSE 7T; FOV: 180 x 180 x 9.75 mm; 6 slices;0.70 x 0.87 x 1.5 mm; slice gap: 0.15 mm; TR: 500 ms; TE: 10 ms; flip angle 90^o^; acquisition matrix: 265 x 204; transverse orientation; TSE factor 3; TA: 6 min 54 s).A TSE scan with higher spatial resolution was locally optimized (HR-TSE 7T; FOV: 180 x 180 x 13 mm; 12 slices acquired in 4 interleaved packages; slice gap: 0 mm; 0.44 x 0.44 x 2 mm; TR: 550 ms; TE: 8.4 ms; flip angle 90^o^; acquisition matrix: 400 x 400; TA: 14 min 47 s).

**Fig 1:**
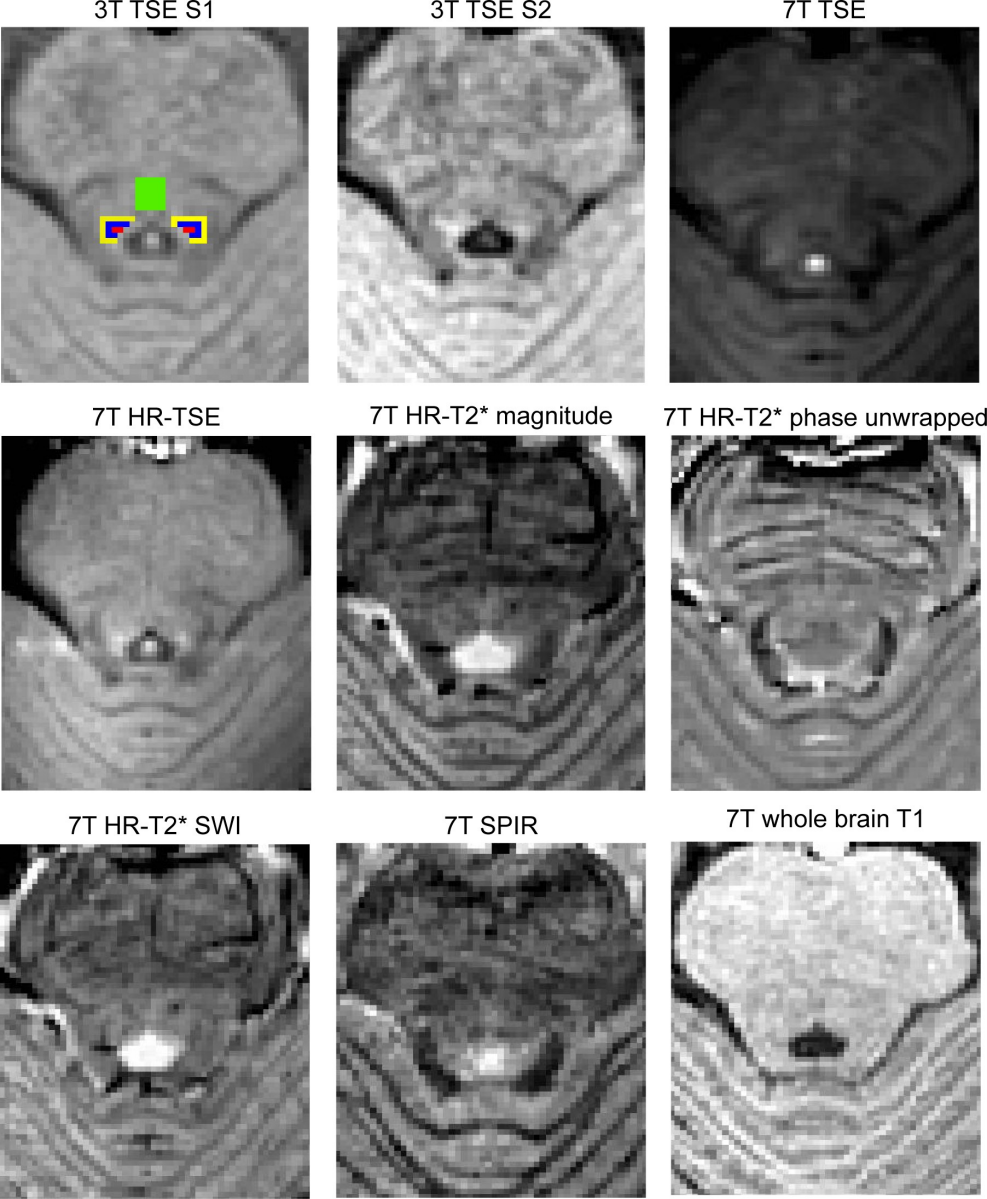
Visual representation of the contrast as obtained from the different scan sequences for a representative participant. The LC masks obtained from the 3T TSE scan in session 1 (S1) are depicted in red (masks of two raters were combined). Blue color indicates voxels that are directly adjacent to the LC mask (plus1). Yellow indicates voxels located two voxels away from the LC mask (plus2). Green color refers to the pontine tegmantum ROI (pt). The other panels show the other eight sequences acquired in this study. All scans were linearly registered to the 7T whole-brain T_1_ scan to enable visualization of the same brain region. The contrast levels for each sequence were normalized using the quickLUT tool in MIPAV. This tool was used to set the image contrast based on the highest and lowest values in an identical region across sequences.

Finally, we obtained a high-resolution T_2_*-weighted scan (HR-T_2_*; FOV: 240 x 180 x 50 mm; 46 slices; 0.23 x 0.23 x 1 mm; slice gap: 0.1 mm; TR: 1766 ms; TE: 25.2 ms; flip angle 60^o^; transverse orientation; acquisition matrix: 1000 x 750; TA: 10 min 09 s) and a T1-weighted SPIR scan (FOV: 180 x 180 x 7.2 mm; 12 slices; 0.60 x 0.60 x 0.60 mm; TR: 9.9 ms; TE: 5.3 ms; flip angle 35^o^; transverse orientation; acquisition matrix: 300 x 294; offset frequency: 1053 Hz; pulse magnitude: 6.17 mT; TA: 7 min 16 s). Total scan duration was approximately 60 minutes. All 3T and 7T sequences were acquired in transverse direction. We chose not to match the sequences on resolution or FOV because, if we would have chosen the lowest common denominator across the sequences, this would have severely limited the capabilities of a number of sequences. In the end, we chose specific, optimal, scan parameters per sequence which have been shown to be successful in depicting subcortical structures. See Table 1 for a summary of the main scan parameters and Fig 1 for an overview of the different contrasts.

**Table 1:** Overview of the scan parameters used to extract the LC contrast.

MR parameter	TSE 3T	TSE 7T	HR-TSE 7T	HR-T_2_* - magnitude	HR-T_2_* - phase unwrapped	HR-T_2_*—SWI	SPIR	Whole-brain T_1_
Field strength	3 T	7 T	7 T	7 T	7 T	7 T	7 T	7 T
Acq. Res.	0.70x0.88x1.5	0.70x0.87x1.5	0.44x0.44x2	0.23x0.23x1	0.23x0.23x1	0.23x0.23x1	0.6x0.6x0.6	0.69x0.69x0.69
Rec. Res.	0.35x0.35x1.5	0.35x0.35x1.65	0.35x0.35x1.0	0.23x0.23x1	0.23x0.23x1	0.23x0.23x1	0.56x0.56x0.6	0.64x0.64x0.69
TE	10 ms	10 ms	8.4 ms	25.2 ms	25.2 ms	25.2 ms	5.3 ms	2.0 ms
TR	500 ms	500 ms	550 ms	1766 ms	1767ms	1767ms	9.9 ms	4.5 ms
Flip angle	90^o^	90^o^	90^o^	60^o^	60^o^	60^o^	35^o^	7^o^
Matrix size	256 x 204	265 x 204	400 x 400	1000 x 750	1000 x 750	1000 x 750	300 x 294	352 x 353
Number of slices	14	6	12	46	46	46	12	249
Acquisition time	7min	6min54s	14min47s	10min09s	10min09s	10min09s	7min16s	8min26s
Total number of subjects	12	12	12	12	11	11	12	12

All DICOM scans were exported to NIfTI using dcm2nii as implemented in MRIcron (V. 2014; [33]).Due to technical reasons the HR-T_2_* unwrapped-phase and SWI scans are missing for one participant. Acq. Res: acquired resolution; Rec. Res: Reconstructed resolution; TE: echo time; TR: repetition time.

### Selection of field of view (FOV) for the slab scans

The field of view (FOV) for the slab acquisitions was selected by two authors (KDT and MvO) after training by a neuroanatomist, extensive piloting and consultation of the literature. During the 3T acquisition the FOV was set perpendicular to the LC and covered the entire LC region (see Tona et al; [19]). For the 7T acquisition the same experts selected the FOV based not only on knowledge about LC position and anatomy but also by having the previously acquired 3T LC scan (of the same participant) as well as its planning relative to the survey scans available at the MRI console during the planning procedure. In this manner, maximum consistency was achieved for the LC localization between and within subjects but also between scanning sessions and different scans.

### Extent of the LC captured by the slab scans

Due to the SAR limitation, the 7T slab scans can suffer from a limited coverage. The priority of this study was to achieve higher contrast and resolution in order to visualize the LC. This came at the expense of brain coverage and the ability to capture the entire rostrocaudal extent of the LC. Specifically, the average percentage of the rostrocaudal extent of the LC (compared to the 3T TSE manual delineation) was 84% (SD 13%) for the 7T TSE sequence, 93% (SD 15%) for the 7T HR-TSE sequence, 100% for the 7T HR T2* slabs, and 60% (SD 27%) for the 7T SPIR sequence.

### Registration procedures

#### Registration of 3T to 7T dataset

All registration steps were done on a single-subject level and performed using FSL (5.0.8.; [34]).The slab of 3T TSE imaging slices was linearly registered to the 7T T_1_-weighted whole-brain volume using FLIRT by means of mutual information, 6 degrees of freedom, and trilinear interpolation. This was done for both 3T TSE volumes. Subsequently, the LC mask of the first scan session at 3T (manually segmented twice by two raters, i.e. 4 segmentations in total) was registered to the 7T whole-brain scan by using the transformation matrix and nearest neighbour interpolation. The 3T LC masks of the first 3T TSE scan session (2 raters X 2 segmentation sessions) were combined and thresholded to create a conjunction mask that contained only voxels that were identified as LC in 3 out of the 4 masks. This conjunction mask was consecutively used as an ROI for measuring the contrast of the LC for all 7T scans, as well as for the second scan session at 3T. Next, two additional masks were created by expanding the LC masks with plus 1 and plus 2 voxels using a 2D box kernel, while excluding the LC mask. Finally, the fourth ventricle was manually segmented on the 3T TSE scan in native space of the first scan session. This was done to ensure that none of the employed masks included any voxels located within the fourth ventricle. Finally, two additional masks were created by expanding the LC masks with plus 1 and plus 2 voxels using a 2D box kernel, while excluding the LC mask. To provide comparable contrast with previous work [4, 35, 36] an additional control region was created in the pontine tegmentum. For all axial slices that contained the LC mask, a region of approximately 4.0 x 4.0 mm was identified in the center of the pontine tegmentum. The resulting LC and control region ROIs can be seen in Fig 1.

#### Registration of 7T dataset

The 7T scans were co-registered to the 7T whole-brain scan using 6 degrees of freedom, mutual information, and trilinear interpolation, except for the 7T TSE scan, for which mutual information failed and normalized correlation was used instead. All 3T and 7T registrations were visually inspected in FSLview by checking the following landmarks for alignment: fourth ventricle floor, the top indentation of the pons, and the bilateral cerebellar superior peduncle. See S1 Fig for examples of the registration accuracy.

## Analysis

### Signal extraction

To measure the contrast of the LC, not only the signal intensity within the LC mask was determined, but also the signal intensity in its immediate surroundings. In more detail, for each image registered to the 7T whole-brain T1-weighted image, we extracted the median signal within the LC ROI, the median signal within the voxels directly adjacent to the LC mask (LC plus 1), and the median signal within the region that is two voxels away from the LC mask (LC plus 2). We also extracted the median signal from the pt ROI.

We included the plus 1 and plus 2 control regions as these ROIs reflect the borders that are of direct interest when parcellating the LC. The reason why we include two different control regions adjacent to the LC (plus 1 and plus 2) was to control for partial volume effects. Given the underlying anatomy of the LC, which has a dense core of neurons and then a diluted area around it [14, 37, 38], it is likely that the directly adjacent voxels (plus 1) still contain parts of the LC.

The pontine tegmentum control region was included because it facilitates comparisons with prior literature and because this ROI is not sensitive to LC-partial volume effects. However, it should be noted that this pons control ROI is not homogenous as it encompasses elements of the pontine reticular formation (i.e., the nucleus raphe pontines and nucleus reticularis tegmentipontis). In addition, the pons control ROI contains both longitudinally and transversely oriented fibre tracts (the tectospinal and decussating trigemino-thalamic tracts respectively; see, e.g., brain atlases such as [39] and [40]).

In our main analysis we registered all the LC masks and MRI sequences to the corresponding whole brain 7T T_1_-weighted image. The advantage of this approach is that it allows us to compare the exact same locations across the different sequences. A limitation however is that it entails a registration step which results in implicit smoothing. Therefore, in addition to the main analysis, the LC masks were projected to each individual sequence native space using the combined transformation matrices.

### Contrast ratio estimation

The contrast of the LC was calculated per hemisphere based on the following relative contrast formulas:

$$LC\;contrast\;1=\frac{LC-LC\;plus\;1}{LC\;plus\;1}*100$$

$$LC\;contrast\;2=\frac{LC-LC\;plus\;2}{LC\;plus\;2}*100$$

$$LC\;contrast\;pt=\frac{LC-PT}{PT}*100$$

where *LC* is the median intensity within the LC ROI, *LC plus 1* is the median intensity in the control region that is one voxel adjacent to the LC ROI, *LC plus 2* is the median intensity in the control region that is two voxels away from the LC ROI and *PT* is the median intensity in the pontine tegmentum control region. To test whether the left and right LC had similar contrast we calculated the Pearson correlation coefficient per contrast and sequence.

The LC contrasts were first calculated for each hemisphere and then averaged, since we had no a-priori hypothesis regarding hemispheric differences in contrast ratio. This resulted in a total of eight contrast values per subject: one for the reference 3T TSE scan and seven for the 7T scans.

### Data preprocessing and Bayesian statistics

The LC contrast 1 and LC contrast 2 were first calculated per hemisphere separately, and subsequently averaged over hemispheres, resulting in a total of eight contrast values per subject: one for the reference 3T TSE scan and seven for the 7T scans. An outlier rejection criterion of three times the interquartile range was subsequently used to test for outliers.

All sequences were compared in a quantitative statistical manner. Bayesian one-sample t-tests using a Cauchy prior of 0.707 were utilized to quantify whether the LC contrasts differed significantly from zero. The one-sample t-tests were carried out using JASP (JASP Team, 2017). The benefit of using Bayesian statistics is that it allows the quantification of evidence for the null hypothesis (H_0_: the intensity of the LC does not differ from the adjacent voxels) versus the alternative hypothesis (H_1_: the intensity of the LC does differ from the adjacent voxels). We used the labels as proposed by Jeffreys [41] and edited by Wetzels and Wagemakers [42]. Table 2 shows this suggested evidence categorization for the BF_10_, but note that these labels are only used to facilitate the interpretation of the evidence and should not zealously be adhered to.

**Table 2:** Suggested categories for interpreting the Bayes factors.

Bayes factor BF_10_			Interpretation
	>	100	Decisive evidence for H_1_
30	-	100	Very strong evidence for H_1_
10	-	30	Strong evidence for H_1_
3	-	10	Substantial evidence for H_1_
1	-	3	Anecdotal evidence for H_1_
	1		No evidence
1/3	-	1	Anecdotal evidence for H_0_
1/10	-	1/3	Substantial evidence for H_0_
1/30	-	1/10	Strong evidence for H_0_
1/100	-	1/30	Very strong evidence for H_0_
	<	1/100	Decisive evidence for H_0_

The scans for which the one-sample t-tests indicated strong or more (BF_10_ > 10.0) evidence in favor of H_1_ were then statistically compared with each other in a JZS Bayesian mixed effect model with contrast ratio as the dependent variable, contrast level and sequence type as the independent variables, and subjects as the random factor. This analysis and the follow-up post-hoc Bayesian paired t-tests were carried out using the Bayes Factor package [43, 44] as implemented in R (V. 3.2.4; [45]). For the paired t-tests, subjects were excluded if they had a missing value due to outlier rejection in any of the tested scans.

## Results

### Descriptives

**Fig 2:**
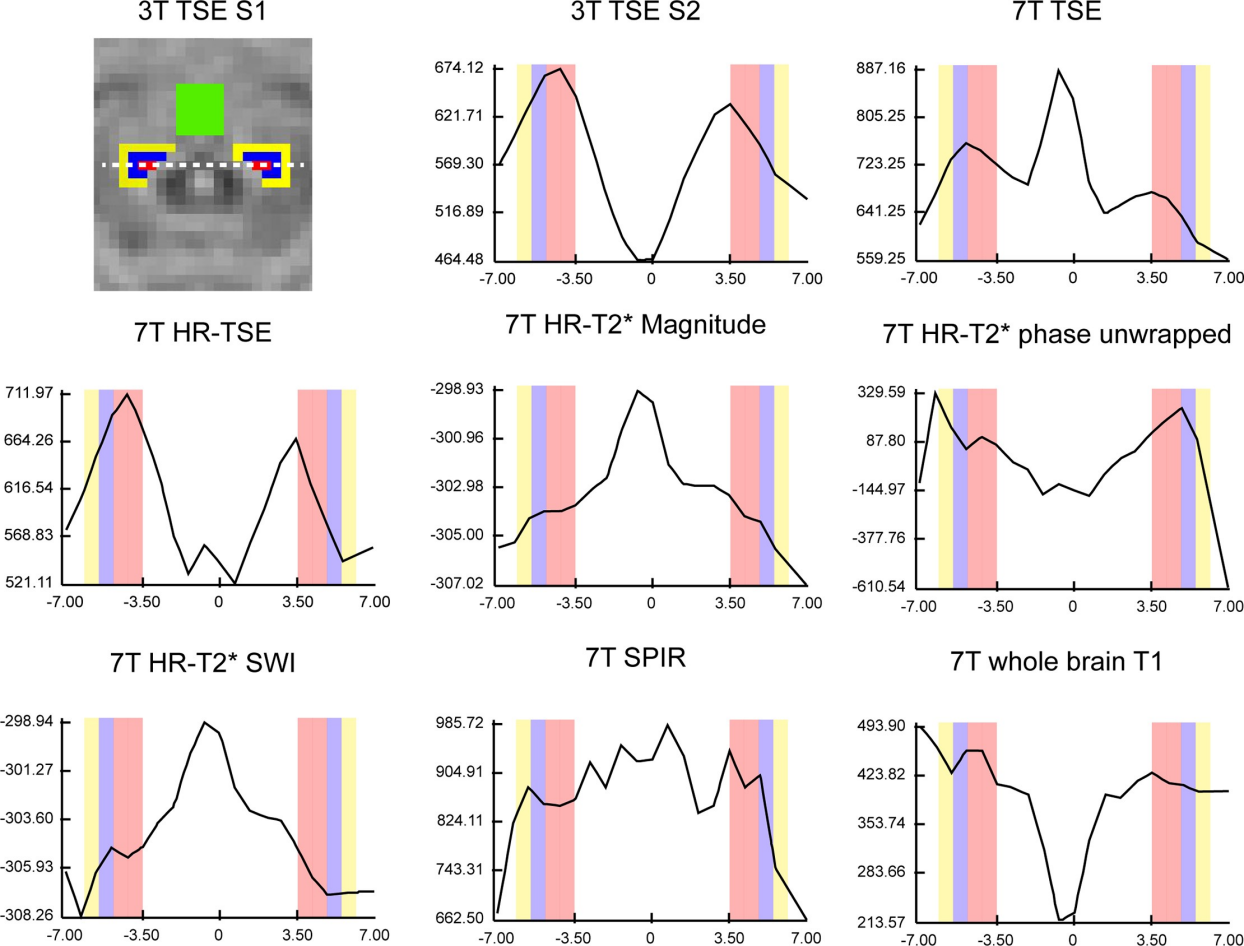
A scan intensity profile figure. The black intensity line shows the change of intensity for a single subject along the dotted line as indicated in the 3T TSE scan of the first session (S1). All intensity values were extracted from the same axial level of the LC across contrasts. The participant and axial level is identical to Fig 1. The point 0 of the x-axis corresponds to the middle of the 4th ventricle, the red bars correspond to the LC mask, the blue and yellow bars correspond respectively to the ROIs of LC plus 1 and LC plus 2. Contrary to this approach (scan intensity profile; which includes only the voxels on the line), the statistical analyses included the median of the entire LC mask, which makes those analyses less susceptible to noise.

After registration of the various images the median signal was extracted for the LC and the surrounding control regions, and the contrasts were calculated. The summary statistics are provided in Table 3. Note that while the 7T HR-T_2_*-magnitude sequence had the smallest contrast, on average it had the highest left-right contrast consistency. Other sequences that had a high left-right contrast consistency were the 7T SPIR and 3T TSE sequence. Also note that depending on the control region, the left-right contrast consistency varied substantially within and between sequences. To visualize the contrast of the different types of scans, a representative subject was selected for whom, for a single slice and profile line, the signal intensity was extracted and plotted (see Fig 2). The descriptive results were very similar when the contrast was extracted in native scan space (see S1 Table and S2 Fig)

**Table 3:** Summary statistics of the estimated LC contrasts for each scan sequence.

		LC contrast 1			LC contrast 2			LC contrast pt		
	N(contrast 1/2/pt)	Avg. median	IQR	Cor. L/R	Avg. median	IQR	Cor. L/R	Avg. median	IQR	Cor. L/R
3T TSE	12/12/12	4.39	1.63	0.68	8.58	1.66	0.47	9.06	2.43	0.47
7T TSE	12/12/12	2.91	3.27	0.50	7.51	3.69	0.48	9.44	9.35	-0.25
7T HR-TSE	12/12/12	2.36	2.32	0.26	5.51	1.46	0.13	6.40	7.18	-0.40
7T HR-T_2_*—magnitude	11/11/9	-0.20	0.16	1.0	-0.36	0.32	1.0	-0.42	0.38	1.0
7T HR-T_2_*—phase unwrapped	11/10/9	-44.72	100.46	0.35	19.92	179.54	0.19	-21.72	150.43	0.74
7T HR-T_2_*—SWI	9/8/7	-0.51	0.39	0.68	0.83	0.53	0.23	1.03	0.71	0.26
7T SPIR	12/12/12	5.60	2.60	0.68	11.90	4.70	0.60	7.49	5.41	0.40
7T whole brain T_1_	12/12/12	1.32	2.77	0.23	-0.66	3.02	-0.15	1.85	4.42	-0.32

N: the number of participants after outlier rejection. Avg. Median: average median. Cor. L/R: Pearson correlation coefficient between the left and right hemisphere contrast. Note that for the T_2_*-based contrasts one participant was missing due to technical reasons; IQR: interquartile range; pt: pontine tegmentum.

### Contrast between the LC and the surrounding tissue

**Fig 3:**
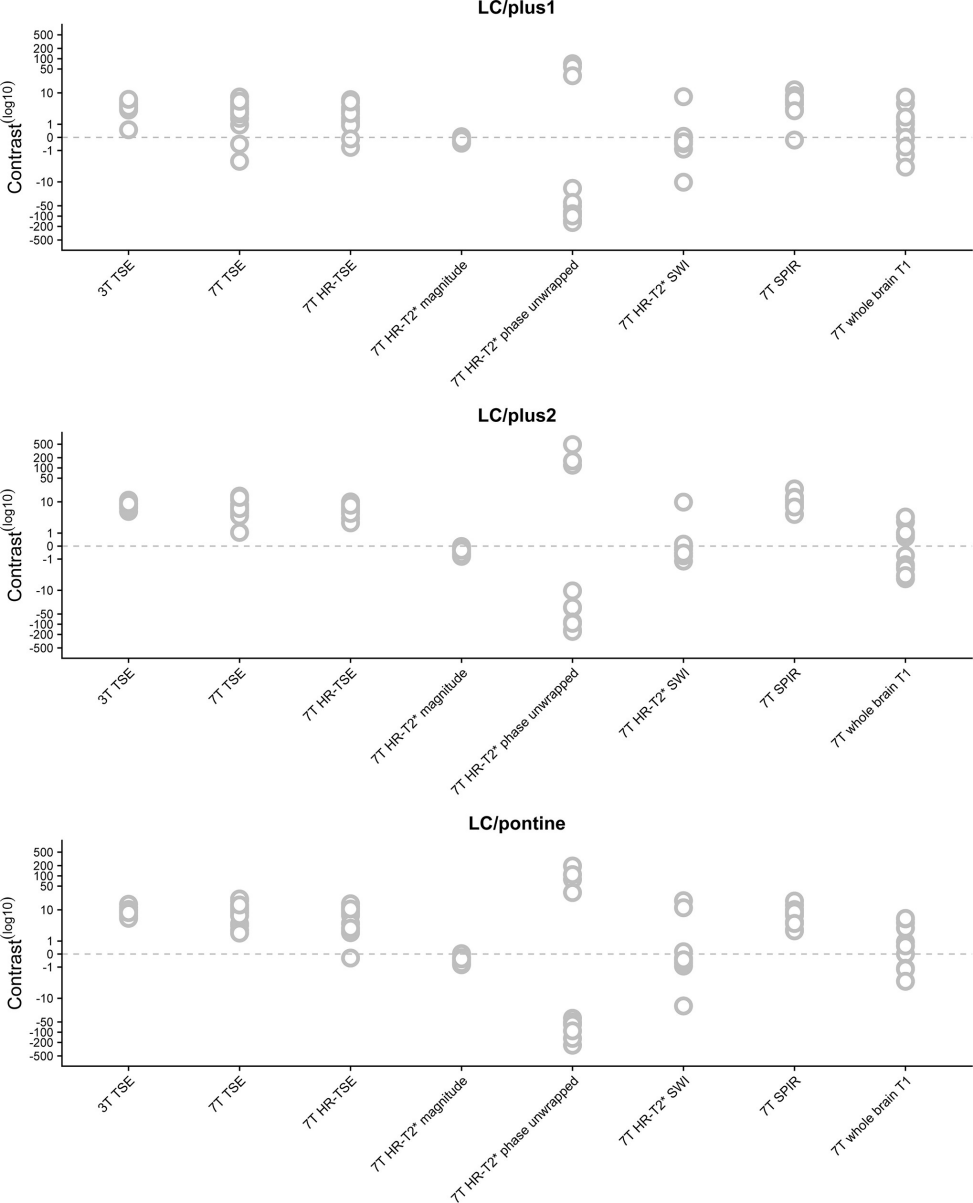
Contrast ratios per scan, separately for each participant. The top panel illustrates the contrast per sequence between the LC and the directly adjacent voxels (LC/plus1). The middle panel illustrates the contrast between the LC and the region which is two voxels removed from the LC (LC/plus2). Finally, in the lower panel, the contrast between the LC and the pontine is shown (LC/pontine). Each circle corresponds to a single subject.

Our first goal was to test whether the different sequences provide any contrast between the LC and the surrounding tissue. To this end, a Bayesian one-sample t-test was used to test whether any of the 8 scan sequences provided a contrast which is different from zero. Fig 3 and Table 4 provide the results for all contrast definitions: LC contrast 1, LC contrast 2, and LC contrast PT.

**Table 4:** Results of the Bayesian one-sample t-tests examining which scans deliver a detectable contrast between the LC and surrounding tissue.

Comparison	N(contrast 1/2/pt)	LC contrast 1 BF_10_	LC contrast 2 BF_10_	LC contrast pt BF_10_
3T TSE ≠ 0	12/12/12	13.4e3	2.98e6	1.72e4
7T TSE ≠ 0	12/12/12	12.8	546	118.56
7T HR-TSE ≠ 0	12/12/12	18.4	12.1e3	57.68
7T HR-T2*—magnitude ≠ 0	11/11/11	72.7	86.2	139.18
7T HR-T2*-phase unwrapped ≠ 0	11/10/10	1.24	0.32	0.35
7T HR-T2*—SWI ≠ 0	9/8/10	0.34	0.40	0.33
7T SPIR ≠ 0	11/11/11	183	1.36e3	66.11
7T whole brain T1 ≠ 0	12/12/12	0.70	0.42	1.72

N: the number of participants after outlier rejection.

The reader is referred to Table 2 for an interpretation of the resulting Bayes factor.

Bayes factors indicated strong or more evidence (BF_10_ > 10.0) in favour of the hypothesis that there is a contrast different from zero between the LC and the surrounding tissue for the following sequences: 3T TSE, 7T TSE, 7T HR-TSE,7T HR-T_2_*-magnitude, and the 7T SPIR. All of these scans showed a positive contrast (i.e., higher signal intensity for LC than the surroundings) except for the HR-T_2_*-magnitude sequence, which on average displayed a lower intensity within the LC (see Fig 3 and Table 4). As these five sequences had a BF_10_ higher than 10.0 for all three contrasts, they were included in the mixed effect model to compare the contrast between sequences. For a proper comparison between the contrasts of the different scans, the 7T HR-T_2_* magnitude contrast scores were inverted by multiplying with -1 for all further analyses. The results are very similar when the signal was extracted in native scan space (S2 Table).

### Mixed effect model to compare the sequences

A JZS Bayesian mixed effect model [44, 46] with default prior scales revealed that the model with main effects of scan sequence and contrast ratio, as well as an interaction between those variables, is preferred above the model without the interaction effect by a Bayes factor of 21.02. The data therefore provide strong evidence that the contrast between the LC and the surrounding tissue is influenced by sequence type, distance from the LC, as well as an interaction between the two factors.

**Fig 4:**
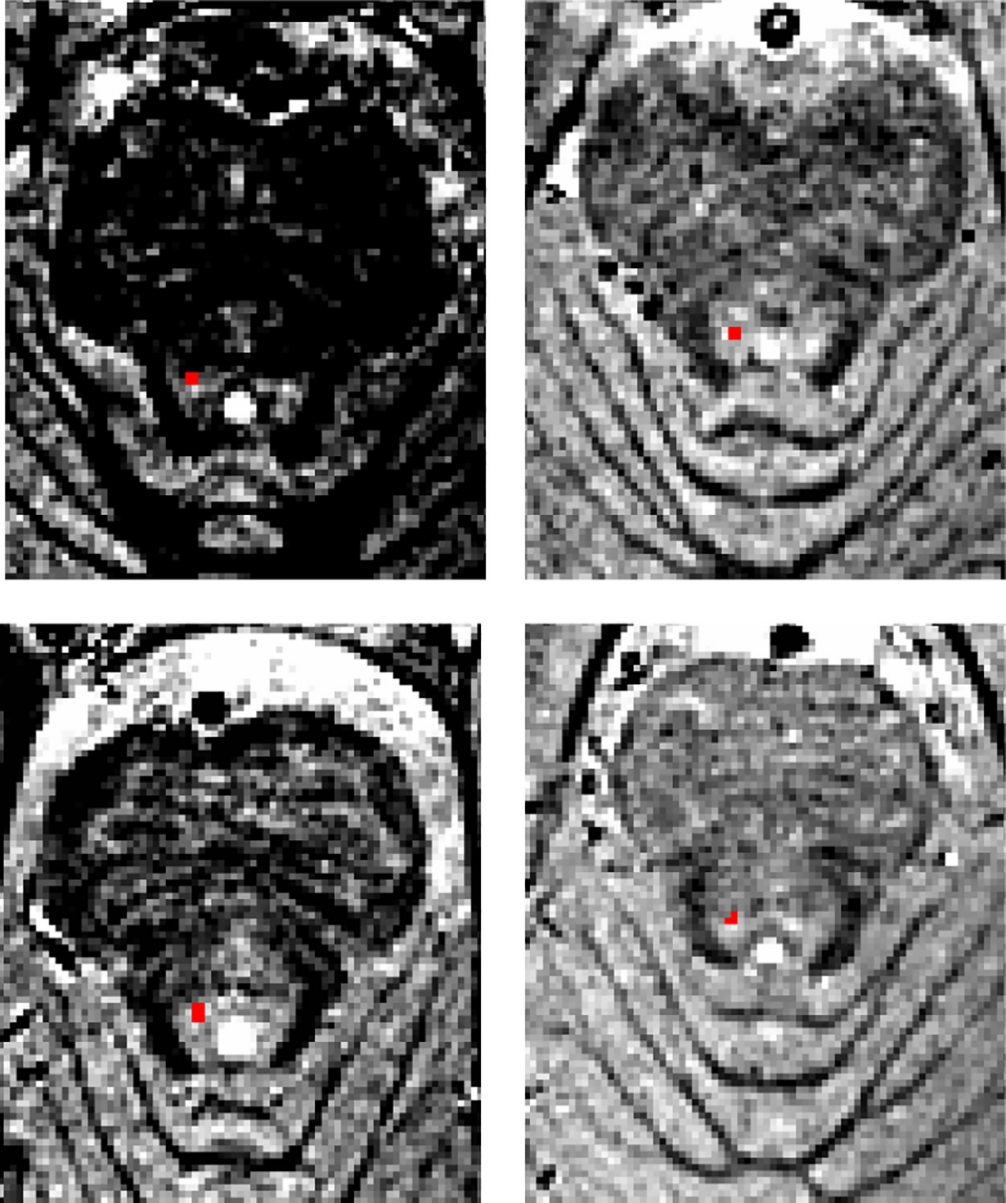
Visualizing the SPIR contrast. For four participants the 7T SPIR volumes in native space are highlighted. The red voxels correspond to the LC as identified on a 3T TSE scan from scan session 1 and registered to the 7T SPIR volume. The contrast levels were set using the min/max values directly surrounding the LC mask.

Post-hoc pairwise comparisons among the five sequences showed decisive evidence that the 7T HR-T_2_* magnitude sequence provides lower contrast than the other four sequences, so this 7T sequence can be discarded (Table 5). The remaining six comparisons yielded three noteworthy findings. First, the results (LC contrast 2) show strong evidence that of the remaining three 7T sequences the SPIR sequence provides higher contrast than the two sequences that were based on the 3T TSE sequence: 7T TSE and 7T-HR TSE. Second, the results of all contrasts provide insufficient evidence (BF_10_ close to 1.0) to determine whether the SPIR sequence has different or similar contrast as the 3T TSE sequence (see Fig 4 for visualization of the SPIR contrast and S3 Fig for the SPIR visualization for all subjects included in the study). Third, and perhaps counterintuitively, we find strong to very strong evidence that the 3T TSE sequence provides higher contrast for the LC with the direct surrounding tissue than the high-resolution 7T TSE sequence. The results are very similar when the signal was extracted in native scan space (S3 Table). This quantitative comparison of contrast was based on the median value of the intensity within the LC masks and control region. However, given that the intensity values within the LC masks may be highly variable, we also provide the (median/(within ROI IQR) ratio within the LC mask for each scan and participant. The results of these extra analyses provide an estimate of “within LC mask”- contrast variability, are in line with the results mentioned here and can be found on S4 Table.

**Table 5:** The post-hoc Bayesian paired t-tests examining whether there was a difference in contrast between sequences, for LC contrast 1, LC contrast 2 and LC contrast pt separately.

Comparison	N	BF_10_ for LC contrast 1	BF_10_ for LC contrast 2	BF_10_ for LC contrast pt
3T TSE– 7T TSE	12	1.19	0.42	0.29
**3T TSE**– 7T HR-TSE	12	15.04*	40.81*	0.85
**3T TSE**– 7T HR-T2* magnitude	11	3547.95*	1.01e+6*	9.7e+4
3T TSE– 7T SPIR	11	0.55	1.98	0.56
7T TSE– 7T HR-TSE	12	0.33	0.90	2.37
**7T TSE**– 7T HR-T2* magnitude	11	6.00	152.59*	63.13
**7T HR-TSE**– 7T HR-T2* magnitude	11	8.10	1326.17*	23.55
**7T SPIR**– 7T HR-T2* magnitude	10	68.09*	422.07*	27.24
**7T SPIR**– 7T TSE	11	1.78	13.33*	0.36
**7T SPIR** –7T HR-TSE	11	2.71	12.76*	0.36

N: the number of participants after outlier rejection. For comparisons with strong evidence for a difference in contrast (*BF_10_ > 10.0), bold-faced sequence has the higher contrast. Pt: pontine tegmentum. The reader is referred to Table 2 for an interpretation of the resulting Bayes factor.

## Discussion

In this study we measured the LC contrast *in vivo* for a number of 7T sequences and compared the obtained contrast measures to a frequently used sequence at 3T. The Bayesian one-sample t-tests indicated that the 3T TSE, 7T TSE, 7T HR-TSE, 7T HR-T_2_*-magnitude, and the 7T SPIR sequences are able to provide significant contrast for visualizing the LC. The statistical analyses also suggested that, of the 7T sequences included in this study, SPIR provides higher contrast than the sequences that were directly based on the 3T TSE sequence. Based on the statistical test, one should conclude that there is not sufficient evidence to state whether the SPIR sequence provides similar, stronger or weaker contrast than the 3T TSE sequence. Further research, preferably with a larger sample size, is needed to address that question. A benefit of the 7T SPIR over the 3T TSE sequence is that, given a similar acquisition time, the 7T sequence has isotropic voxels which are approximately 4 times as small in volume as the voxel size of the 3T TSE scan employed in this study. There are clear advantages of isotropic voxels for edge detection, and therefore volume estimates, of small brain areas [47]. However due to SAR limitations the SPIR scan did not cover the entire brain. This leads to some limitations that we further comment on in the limitation section. Recent efforts by [17] have been successful in visualizing the LC using a MT-weighted FLASH sequence with 0.4 x 0.4 x 0.5mm resolution at 7T, whereas [8] visualized the LC using a T_1_-weighted FLASH sequence with 0.75mm isotropic resolution at 3T. The advantage of those sequences is that they were able to visualize the entire rostrocaudal extent of the LC. Unfortunately, these efforts were unknown when our data were acquired. While the choice of sequences in the current study might be less optimal than the sequences by [17] and [8], our results complement these efforts by directly quantifying the LC contrast with a number of control regions in a Bayesian framework. Additionally, we examined a number of sequences whose utility for LC imaging has been highlighted by prior literature whereas these sequences were not tested by [17] and [8]; e.g. 7T TSE, 7T HR-T_2_*—magnitude, 7T HR-T_2_*-phase unwrapped, 7T HR-T_2_*—SWI. In summary, our study confirms that a MT-weighted (by exploiting the SPIR module in our protocol) FLASH sequence seems the most appropriate method for LC scanning at 7 Tesla.

There has been an ongoing discussion about whether the LC signal intensity should be normalized to a reference region and if so, which region would be most suitable (Liu et al. 2017). Here, we normalized the LC intensity with three different reference regions: the voxels directly adjacent to the LC, two voxels away from the LC mask, and a frequently used area in the pontine tegmentum. The mixed effect model results showed that the contrast between the LC and surrounding tissue is influenced by sequence type, distance from the LC, as well as an interaction between the two factors, suggesting that the LC contrast with the surrounding tissue increases when the control region is not taken directly adjacent to the LC. Given the underlying anatomy of the LC, which has a dense core of neurons, this result can be explained by partial volume effects which are smaller as the distance between the core LC and surrounding tissue increases, and thus result in a larger contrast. For segmentation purposes the LC contrast with the plus 1 and plus 2 reference region seems the most relevant as it entails the border that one wants to identify. The LC contrast with the pontine tegmentum control region seems to be most relevant for comparing our results with previous work (Liu et al. 2017). But a cautionary note should be made, because it is known that the pontine tegmentum shows age-related changes in intensity, which complicates comparisons between LC contrasts normalized with such a reference region over a wide age range [9], [11]. The choice of the control region also has an influence on hemispheric contrast consistency. As shown in Table 3, depending on which control region is used, the correlation coefficient between the left and right LC contrast can vary substantially. This effect of control region should be taken into account when interpreting the various reports on hemisphere difference in LC intensity (e.g., [19], [14], [8], [48]).

### Potential contrast mechanism of 7T SPIR sequence

Of the seven 7T sequences we tested, SPIR was found to give the highest contrast between the LC and the surrounding tissue. Here we discuss the potential underlying contrast mechanism of this sequence. It has been argued that the origin of MRI contrast in neuromelanin imaging is based on the combination of a number of factors: presence of neuromelanin, but also iron and copper, as well as the occurrence of magnetization transfer (MT) effects [14, 17, 49–53]. Although the primary use of a SPIR pulse is to suppress the signal from fat, this pulse does introduce some MT effects into the contrast as the pulse is off-resonance with respect to the water peak [54]. Besides the T_1_ component in the employed SPIR sequence, the MT contribution in combination with T_1_-weighting might be the reason why the SPIR sequence was found to be the most promising sequence for LC imaging at 7T. Indeed, in line with our results, a recently published study shows that MT-weighted sequences can deliver sufficient contrast to visualize the LC *in vivo* at 7T [17]. However, other recent work imaging phantoms with different concentrations of iron and synthetic melanin, indicates that the presence of melanin with or without iron does not lead to direct MT effects [52]. Instead any reduction in MT ratio (MTR) is due to T_1_ effects of the free water pool. Thus, the MTR can be indirectly reduced in the presence of melanin-iron complexes, as a result of T_1_ shortening. Based on the current results as well as previous results [17, 52, 53] it seems likely that MT play an important role in LC imaging, along with other factors that determine the contrast (e.g., T_1_ effects).

### Limitations

This study has a few limitations. The first is that we did not randomize the order of scan sequences over the subjects, which might have resulted in more motion artifacts in the later scans [55]. However, it should be noted that the 7T SPIR sequence, which was the most promising 7T sequence, was always acquired last. Additionally it is important to state that the employed sequences in this study or in literature are mainly sensitive but not exclusively sensitive to neuromelanin. Contrast in the scans is therefore likely to differ between sequences as well as between field strengths. Another limitation pertains to the choice of 6 degrees of freedom for all registration steps. The rationale for this choice was that all registration steps were done in a “within-subject” fashion (inter-subject analysis) and that we did not want to incorporate different scaling factors for registration analysis applied to different datasets. While all registration steps were visually inspected and several examples of registration accuracy are given in the S1 Fig, it might still be the case that different geometric distortions between field strengths resulted in subtle misalignments (see for instance [56]). As a result, due to the concatenation of two 3T towards 7T transformation matrices, it might explain why the number of outliers for the 3T TSE sequence in native space is higher than in 7T T_1_-space. Another limitation is that we did not acquire quantitative MRI (qMRI) sequences. There are several benefits of qMRI (T_1_, T_2_, T_2_*, PD, MT etc) over standard weighted sequences as the values are standardized and allow for direct comparison between centers [57]. Another advantage of qMRI is that a physical meaning can be assigned to the intensity of the voxel, which can then be linked to the underlying mechanism contributing to the contrast [58]. Contrary to the SPIR sequence, which was not optimized to maximize MT effects, the acquisition of qMT volumes would have allowed for a direct quantification of the contribution of MT to the LC contrast. Another limitation is that the CNR comparisons were only performed on young participants. It is known that the level of neuromelanin in young adults is lower than in elderly [59, 60]. Such age-related changes in the tissue property might influence the generalizability of our results and should be considered when including different age groups.

It should also be noted that the recently published sequences by Priovoulos et al. (includes 7T sequences) and Betts et al. (includes 3T sequences) were not included in our set of sequences, since these studies were only published after our data collection. Sequences in our protocol resembled their settings, but were slightly different. Moreover, our results complement their efforts by directly quantifying the LC contrast with a number of control regions in a Bayesian framework. Additionally, we examined a number of sequences whose utility for LC imaging has been suggested by prior literature but were not tested by Priovoulos and Betts; e.g. 7T TSE, 7T HR-T_2_*—magnitude, 7T HR-T_2_*-phase unwrapped, 7T HR-T_2_*—SWI. In summary, our study confirms arguments by Priovoulos et al. and Betts et al. that a MT-weighted (by exploiting the SPIR module in our protocol) FLASH sequence seems the most appropriate method for LC scanning at 7 Tesla.

Additionally, while all registration steps were visually inspected and several examples are given in the S1 Fig, it might still be the case that different geometric distortions between field strengths resulted in subtle misalignments (see for instance [56]). As a result, due to the concatenation of two 3T towards 7T transformation matrices, it might explain why the number of outliers for the 3T TSE sequence in native space is higher than in 7T T_1_-space.

### Suggestions for future directions

Future studies should further optimize the SPIR sequence to allow for manual segmentation of the LC and improve the coverage of the rostrocaudal extent of the LC, for example with multiband MRI. Another possibility that future studies might consider is to potentially increase the SPIR contrast by modifying the suppression pulse amplitude, the off-resonance frequency and time between suppression and excitation. Additionally, some of the SNRs for the 7T images are lower than the 3T (see S4 Table), which might indicate that the increase in resolution costs more SNR than is gained by moving to 7T. Therefore, future studies comparing 3T with 7T could incorporate a design where the SNR is kept constant between the two modalities by finding the exact resolution on the 7T that would match the SNR at 3T and then compare the detectability of the LC.

## Conclusion

In conclusion, by quantitatively comparing the contrast of the LC with the surrounding tissue for a number of sequences, we have identified a promising SPIR-based sequence. This sequence provides similar contrast of the LC as the 3T sequence commonly used, but at a higher spatial resolution (compared to the 3T TSE scan used in the majority of studies) and with isotropic voxels. The isotropic voxels at 7T should be considered an important advantage given the small size of the LC. Finally, although there is no clear benefit in contrast, a potential advantage of using SPIR is the relatively short acquisition time, which may be desirable in clinical settings to minimize subject motion.

## Supporting information

S1 Table Summary statistics of the estimated LC contrasts for each scan sequence in native scan space. N: the number of participants after outlier rejection, note that for the T_2_* based contrasts one participant was missing due to technical reasons; IQR: interquartile range; pt: pontine tegmentum.
(DOCX)

S2 Table Results of the Bayesian one-sample t-tests examining which scans deliver a detectable contrast between the LC and surrounding tissue. Contrast is extracted in native scanner space. N: the number of participants after outlier rejection. Similar to the results in 7T T_1_ space, the Bayesian one-sample t-tests indicate strong or more evidence (BF10>10.0) in favour of the hypothesis that there is a contrast different from zero between the LC and the surrounding tissue for the 3T TSE, 7T TSE, 7T HR-TSE, 7T HR-T_2_*-magnitude, and the 7T SPIR. Statistically significant values (BF10>10.0) are also indicated by asterisk “*”.
(DOCX)

S3 Table The post-hoc Bayesian paired t-tests examining whether there was a difference in contrast between sequences, for LC contrast 1 and LC contrast 2 separately. N: the number of participants after outlier rejection. The Bayesian paired t-tests indicate strong or more evidence (BF10>10.0) in favour of the hypothesis that there is a difference in contrast between sequences for LC contrast 1 and LC contrast 2 separately. Statistically significant values (BF10>10.0) are also indicated by asterisk “*”.
(DOCX)

S4 Table Within LC mask contrast variability. The quantitative comparison of contrast was based on the median value of the intensity within the LC masks and control region. However, given that the intensity values within the LC masks may be highly variable, we also provide the (median/(within ROI IQR) ratio within the LC mask for each scan and participant. Results are reported separately for each hemisphere. Analysis was done in native space. “–”indicates missing data either due to FoV placement (SPIR) or technical reasons (SWI, phase unwrapped).
(DOCX)

S1 Fig Registration accuracy of the different sequences to 7T T_1_ whole brain. As can be noted by the skull contour, sulci patterns and borders of the 4^th^ ventricle, the registration employing 6 DoF was successful between the different sequences and the 7T T_1_ whole brain volume. The yellow pixels directly adjunct to the 4^th^ ventricle correspond to the locus coeruleus in the left hemisphere.
(DOCX)

S2 Fig Contrast ratios per scan in native scan space, separately for each participant. (DOCX)

S3 Fig Depiction of 7T SPIR sequence in native space for each participant separately. For all participants, an axial slice of the 7T SPIR sequence is shown in native space. The red voxels correspond to the left LC as identified on a 3T TSE scan from scan session 1 and registered to the 7T SPIR volume. The contrast levels were set using the min/max values directly surrounding the LC mask. The arrow indicates the location of the right LC.
(DOCX)
