# Supplementary material for: Quantifying the contrast of the human locus coeruleus in vivo at 7 Tesla MRI
Source: PLoS One. 2019 Feb 6;14(2):e0209842. doi: 10.1371/journal.pone.0209842 (PMC6364884; doi:10.1371/journal.pone.0209842)
Supplement: S1 Table — N: the number of participants after outlier rejection, note that for the T2* based contrasts one participant was missing due to technical reasons; IQR: interquartile range; pt: pontine tegmentum. (DOCX) [file pone.0209842.s001.docx]

In the main text the LC masks and MRI sequences were all registered to the corresponding 7T T_1_-weighted image. This registration step does however result in implicit smoothing and partial voluming effects. Therefore, in addition to the main analysis, the LC masks were projected to each individual sequence native space using the combined transformation matrices. As is shown below (S1 and S2 Tables; S2 Fig.), the results in native space are very similar to the results in 7T T_1_ space.

**S1 Table. Summary statistics of the estimated LC contrasts for each scan sequence in native scan space.**

|  |  | | LC contrast 1 | | | LC contrast 2 | | | | | | LC contrast PT | | | | |
| --- | --- | --- | --- | --- | --- | --- | --- | --- | --- | --- | --- | --- | --- | --- | --- | --- |
|  | N(contrast 1/2/pt) | | Average median | | IQR | Average median | | IQR | | Average median | | | IQR | |  |  |
| 3T TSE | 12/10/11 | 6.49 | | 2.08 | | | 11.04 | | 0.91 | | 10.62 | | | 2.6 | |  |
| 7T TSE | 12/12/12 | 3.01 | | 4.31 | | | 8.06 | | 4.47 | | 10.46 | | | 8.11 | |  |
| 7T HR-TSE | 12/12/12 | 3.39 | | 4.36 | | | 6.46 | | 3.47 | | 7.68 | | | 8.39 | |  |
| 7T HR-T_2_* - magnitude | 11/11/11 | -0.21 | | 0.13 | | | -0.38 | | 0.26 | | -0.46 | | | 0.31 | |  |
| 7T HR-T_2_* - phase unwrapped | 11/11/11 | -60.85 | | 118.63 | | | 22.6 | | 262.09 | | -99.2 | | | 231.40 | |  |
| 7T HR-T_2_* - SWI | 10/8/9 | 1.75 | | 0.48 | | | 0.93 | | 0.71 | | 3.69 | | | 0.75 | |  |
| 7T SPIR | 11/11/11 | 6.02 | | 2.5 | | | 12.23 | | 6.38 | | 7.65 | | | 4.42 | |  |
| 7T whole brain T_1_ | 12/12/12 | 1.32 | | 2.77 | | | -0.66 | | 3.02 | | 1.85 | | | 4.42 | |  |

*N: the number of participants after outlier rejection, note that for the T_2_* based contrasts one participant was missing due to technical reasons; IQR: interquartile range; pt: pontine tegmentum.*
