# Supplementary material for: Quantifying the contrast of the human locus coeruleus in vivo at 7 Tesla MRI
Source: PLoS One. 2019 Feb 6;14(2):e0209842. doi: 10.1371/journal.pone.0209842 (PMC6364884; doi:10.1371/journal.pone.0209842)
Supplement: S2 Table — Contrast is extracted in native scanner space. N: the number of participants after outlier rejection. Similar to the results in 7T T1 space, the Bayesian one-sample t-tests indicate strong or more evidence (BF10>10.0) in favour of the hypothesis that there is a contrast different from zero between the LC and the surrounding tissue for the 3T TSE, 7T TSE, 7T HR-TSE, 7T HR-T2*-magnitude, and the 7T SPIR. Statistically significant values (BF10>10.0) are also indicated by asterisk “*”. (DOCX) [file pone.0209842.s002.docx]

**S2 Table. Results of the Bayesian one-sample t-tests examining which scans deliver a detectable contrast between the LC and surrounding tissue. Contrast is extracted in native scanner space.**

| Comparison | N(contrast 1/2/pt) | LC contrast 1 BF_10_ | LC contrast 2 BF_10_ | LC contrast PT BF_10_ |
| --- | --- | --- | --- | --- |
| 3T TSE ≠ 0 | 12/10/11 | 3372.54 ^*^ | 460533.1 ^*^ | 925874.1 ^*^ |
| 7T TSE ≠ 0 | 12/12/12 | 16.28 ^*^ | 5060.3 ^*^ | 235.62 ^*^ |
| 7T HR-TSE ≠ 0 | 12/12/12 | 16.16 ^*^ | 6232.48 ^*^ | 60.04 ^*^ |
| 7T HR-T_2_* - magnitude ≠ 0 | 11/11/11 | 55.74 ^*^ | 84.99 ^*^ | 178.52 ^*^ |
| 7T HR-T_2_*-phase unwrapped ≠ 0 | 11/11/11 | 3.06 | 0.31 | 0.54 |
| 7T HR-T_2_* - SWI ≠ 0 | 10/8/9 | 0.38 | 0.4 | 0.6 |
| 7T SPIR ≠ 0 | 11/11/11 | 2363.7 ^*^ | 1687.39 ^*^ | 159.27 ^*^ |
| 7T whole brain T_1_ ≠ 0 | 12/12/12 | 0.7 | 0.42 | 1.72 |

*N: the number of participants after outlier rejection.*

Similar to the results in 7T T_1_ space, the Bayesian one-sample t-tests indicate strong or more evidence (BF10>10.0) in favour of the hypothesis that there is a contrast different from zero between the LC and the surrounding tissue for the 3T TSE, 7T TSE, 7T HR-TSE, 7T HR-T_2_*-magnitude, and the 7T SPIR. Statistically significant values (BF10>10.0) are also indicated by asterisk “^*”^.

A JZS Bayesian mixed effect model (Morey & Rouder, 2015; Rouder et al., 2012) with default prior scales revealed that the model with main effects of scan sequence and contrast ratio, as well as an interaction between those variables, is preferred above the model without the interaction effect by a Bayes factor of 55.10. The data therefore provide very strong evidence that the contrast between the LC and the surrounding tissue is influenced by sequence type, control region, as well as an interaction between the two factors.
