# Supplementary material for: Quantifying the contrast of the human locus coeruleus in vivo at 7 Tesla MRI
Source: PLoS One. 2019 Feb 6;14(2):e0209842. doi: 10.1371/journal.pone.0209842 (PMC6364884; doi:10.1371/journal.pone.0209842)
Supplement: S3 Table — N: the number of participants after outlier rejection. The Bayesian paired t-tests indicate strong or more evidence (BF10>10.0) in favour of the hypothesis that there is a difference in contrast between sequences for LC contrast 1 and LC contrast 2 separately. Statistically significant values (BF10>10.0) are also indicated by asterisk “*”. (DOCX) [file pone.0209842.s003.docx]

Post-hoc pairwise comparisons among the five sequences showed that, similar to the results in 7T T_1_ space, the T_2_*-magnitude image provides lower contrast that the other four sequences (S3 Table). The overall pattern of the remaining results is very similar to the results obtained in 7T T_1_ space (Table 5 versus S3 Table).

**S3 Table. The post-hoc Bayesian paired t-tests examining whether there was a difference in contrast between sequences, for LC contrast 1 and LC contrast 2 separately.**

| Comparison | N | BF_10_ for LC contrast 1 | BF_10_ for LC contrast 2 | BF_10_ for LC contrast pt |
| --- | --- | --- | --- | --- |
| 3T TSE – 7T TSE | 9 | 18.16 ^*^ | 2.14 | 0.33 |
| 3T TSE – 7T HR-TSE | 9 | 43.61 ^*^ | 41.95 ^*^ | 0.7 |
| 3T TSE – 7T HR-T_2_* magnitude | 8 | 2620.32 ^*^ | 250573.9 ^*^ | 110713.3 ^*^ |
| 3T TSE – 7T SPIR | 8 | 0.51 | 0.36 | 30.92 ^*^ |
| 7T TSE – 7T HR-TSE | 12 | 0.30 | 0.58 | 1.74 |
| 7T TSE – 7T HR-T_2_* magnitude | 11 | 8.71 | 1190.95 ^*^ | 179.98 ^*^ |
| 7T HR-TSE – 7T HR-T_2_* magnitude | 11 | 8.82 | 784.67 ^*^ | 20.51 ^*^ |
| 7T SPIR – 7T HR-T_2_* magnitude | 10 | 739.33 ^*^ | 503.21 ^*^ | 53.22 ^*^ |
| 7T SPIR – 7T TSE | 11 | 2.14 | 6.54 | 0.52 |
| 7T SPIR – 7T HR-TSE | 11 | 1.19 | 4.15 | 0.30 |
|  |  |  |  |  |

*N: the number of participants after outlier rejection.*

The Bayesian paired t-tests indicate strong or more evidence (BF10>10.0) in favour of the hypothesis that there is a difference in contrast between sequences for LC contrast 1 and LC contrast 2 separately. Statistically significant values (BF10>10.0) are also indicated by asterisk “^*”^.
