# Supplementary material for: Quantifying the contrast of the human locus coeruleus in vivo at 7 Tesla MRI
Source: PLoS One. 2019 Feb 6;14(2):e0209842. doi: 10.1371/journal.pone.0209842 (PMC6364884; doi:10.1371/journal.pone.0209842)
Supplement: S4 Table — The quantitative comparison of contrast was based on the median value of the intensity within the LC masks and control region. However, given that the intensity values within the LC masks may be highly variable, we also provide the (median/(within ROI IQR) ratio within the LC mask for each scan and participant. Results are reported separately for each hemisphere. Analysis was done in native space. “–”indicates missing data either due to FoV placement (SPIR) or technical reasons (SWI, phase unwrapped). (DOCX) [file pone.0209842.s004.docx]

**S4 Table. Within LC mask contrast variability.** *The quantitative comparison of contrast was based on the median value of the intensity within the LC masks and control region. However, given that the intensity values within the LC masks may be highly variable, we also provide the (median/within ROI IQR) ratio within the LC mask for each scan and participant. Results are reported separately for each hemisphere. Analysis was done in native space. “–“ indicates missing data either due to FoV placement (SPIR) or technical reasons (SWI, phase unwrapped).*

| Median/IQR (per hemisphere) | | | | | | | |  |  |  |  |  |  |  |
| --- | --- | --- | --- | --- | --- | --- | --- | --- | --- | --- | --- | --- | --- | --- |
| Scan | Hemisphere | 1 | 2 | 3 | 4 | 5 | 6 | 7 | 8 | 9 | 10 | 11 | 12 | Average |
| 3T TSE | Left | 15.6 | 9.6 | 12.4 | 10.6 | 15.2 | 20.9 | 19.2 | 18.1 | 21.4 | 19.2 | 17.4 | 11.6 | 15.9 |
|  | Right | 15.8 | 12.4 | 14.0 | 12.4 | 22.9 | 17.6 | 12.8 | 12.1 | 17.6 | 11.5 | 19.0 | 17.1 | 15.4 |
|  |  |  |  |  |  |  |  |  |  |  |  |  |  |  |
| 7T TSE | Left | 9.9 | 5.7 | 8.7 | 10.3 | 14.1 | 21.1 | 12.2 | 12.4 | 12.2 | 16.8 | 10.6 | 8.8 | 11.9 |
|  | Right | 17.9 | 13.7 | 10.5 | 17.0 | 25.2 | 28.2 | 19.0 | 17.7 | 7.1 | 8.6 | 28.9 | 8.5 | 16.9 |
|  |  |  |  |  |  |  |  |  |  |  |  |  |  |  |
| 7T HR-TSE | Left | 7.0 | 8.8 | 10.0 | 5.6 | 11.1 | 13.4 | 6.6 | 11.5 | 14.5 | 16.2 | 8.7 | 10.4 | 10.3 |
|  | Right | 7.7 | 12.9 | 12.8 | 7.0 | 7.4 | 9.2 | 11.8 | 13.4 | 14.3 | 8.9 | 9.5 | 13.6 | 10.7 |
|  |  |  |  |  |  |  |  |  |  |  |  |  |  |  |
| 7T HR-T2* - magnitude | Left | -116.2 | -197.1 | -248.7 | -131.6 | -219.1 | -165.0 | -198.8 | -165.5 | 4.9 | -180.6 | -284.5 | -165.1 | -172.3 |
|  | Right | -131.6 | -132.0 | -220.8 | -123.1 | -246.1 | -197.8 | -198.5 | -198.1 | 6.4 | -219.5 | -180.7 | -165.0 | -167.2 |
|  |  |  |  |  |  |  |  |  |  |  |  |  |  |  |
| 7T HR-T2* - phase unwrapped | Left | 0.2 | -0.1 | 0.5 | 0.4 | -0.5 | 0.5 | 0.2 | 0.5 | 0.1 | -0.1 | 0.0 | - | 0.2 |
|  | Right | 0.8 | 0.6 | 0.5 | 0.0 | -0.2 | 0.1 | 0.2 | 0.6 | -0.3 | -0.1 | -0.4 | - | 0.2 |
|  |  |  |  |  |  |  |  |  |  |  |  |  |  |  |
| 7T HR-T2* - SWI | Left | 0.8 | 7.4 | -124.9 | -76.3 | -219.3 | -110.5 | 5.0 | -117.1 | -110.5 | -221.4 | 1.1 | - | -87.8 |
|  | Right | 0.2 | 3.2 | -142.5 | -94.1 | -151.7 | -132.2 | 4.5 | -153.0 | -197.7 | -153.0 | 2.4 | - | -92.2 |
|  |  |  |  |  |  |  |  |  |  |  |  |  |  |  |
| 7T SPIR | Left | 12.1 | 7.8 | 9.4 | 8.4 | 45.3 | 6.3 | 8.6 | 10.5 | 10.8 | 12.9 | 9.7 | 23.5 | 13.8 |
|  | Right | 10.1 | 6.1 | 5.6 | 10.0 | 5.1 | 12.0 | 7.3 | 7.6 | 9.9 | 14.7 | 7.7 | - | 8.7 |
|  |  |  |  |  |  |  |  |  |  |  |  |  |  |  |
| 7T whole brain T1 | Left | 15.3 | 7.6 | 10.0 | 7.8 | 10.7 | 19.8 | 12.0 | 11.0 | 13.0 | 10.9 | 12.7 | 11.3 | 11.8 |
|  | Right | 11.7 | 11.5 | 16.8 | 5.2 | 6.8 | 12.9 | 8.0 | 11.9 | 12.0 | 12.3 | 10.1 | 6.1 | 10.4 |
