# Supplementary material for: Quantifying the contrast of the human locus coeruleus in vivo at 7 Tesla MRI
Source: PLoS One. 2019 Feb 6;14(2):e0209842. doi: 10.1371/journal.pone.0209842 (PMC6364884; doi:10.1371/journal.pone.0209842)

**S1 Fig. Registration accuracy of the different sequences to 7T T_1_ whole brain.** *As can be noted by the skull contour, sulci patterns and borders of the 4^th^ ventricle, the registration employing 6 DoF was successful between the different sequences and the 7T T_1_ whole brain volume. The yellow pixels directly adjunct to the 4^th^ ventricle correspond to the locus coeruleus in the left hemisphere.*


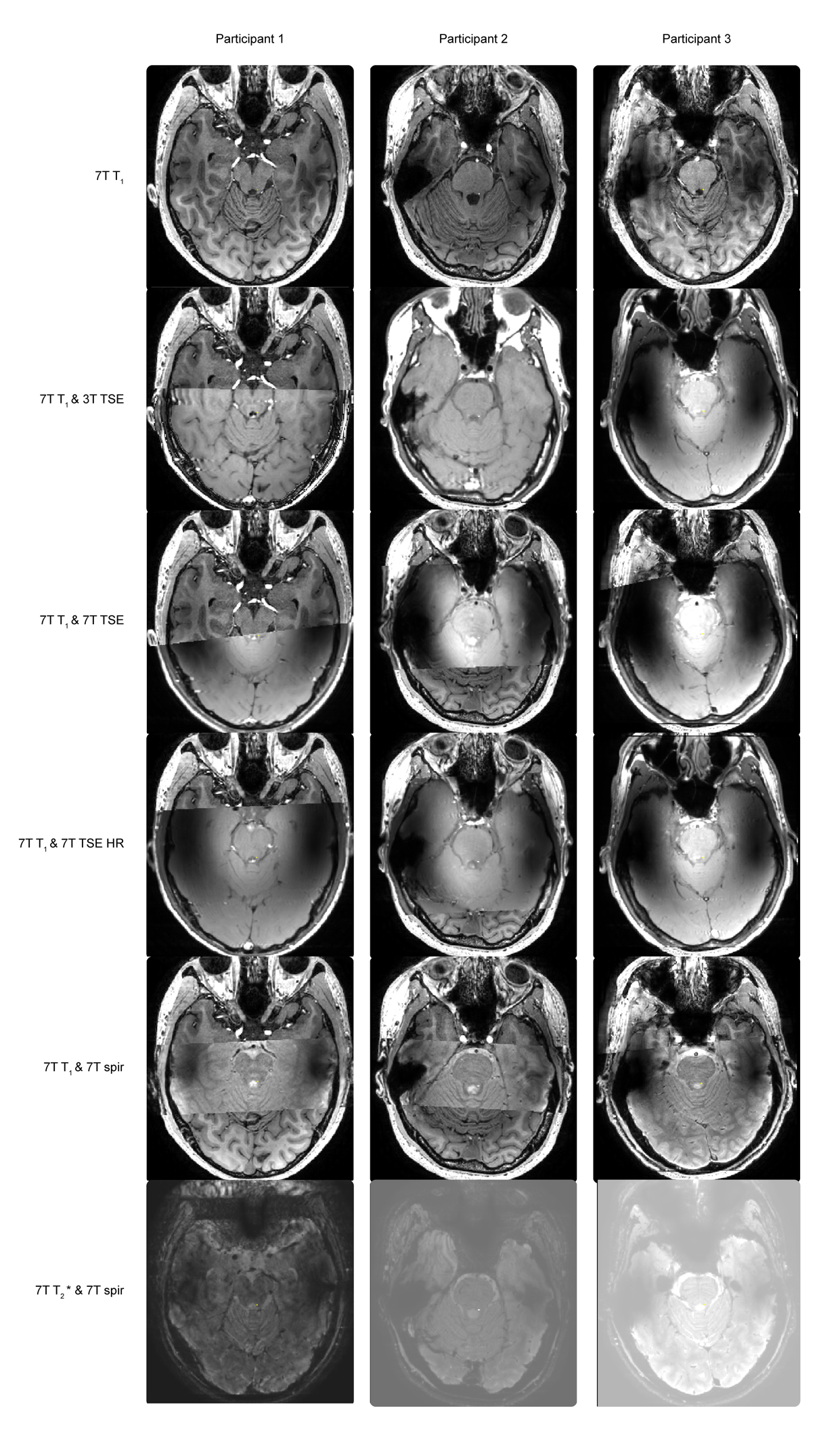

Supplement: S1 Fig — As can be noted by the skull contour, sulci patterns and borders of the 4th ventricle, the registration employing 6 DoF was successful between the different sequences and the 7T T1 whole brain volume. The yellow pixels directly adjunct to the 4th ventricle correspond to the locus coeruleus in the left hemisphere. (DOCX) [file pone.0209842.s005.docx]
