# Supplementary material for: Quantifying the contrast of the human locus coeruleus in vivo at 7 Tesla MRI
Source: PLoS One. 2019 Feb 6;14(2):e0209842. doi: 10.1371/journal.pone.0209842 (PMC6364884; doi:10.1371/journal.pone.0209842)
Supplement: S2 Fig — (DOCX) [file pone.0209842.s006.docx]

**S2 Fig. Contrast ratios per scan in native scan space, separately for each participant.**
