# Supplementary material for: Quantifying the contrast of the human locus coeruleus in vivo at 7 Tesla MRI
Source: PLoS One. 2019 Feb 6;14(2):e0209842. doi: 10.1371/journal.pone.0209842 (PMC6364884; doi:10.1371/journal.pone.0209842)
Supplement: S3 Fig — For all participants, an axial slice of the 7T SPIR sequence is shown in native space. The red voxels correspond to the left LC as identified on a 3T TSE scan from scan session 1 and registered to the 7T SPIR volume. The contrast levels were set using the min/max values directly surrounding the LC mask. The arrow indicates the location of the right LC. (DOCX) [file pone.0209842.s007.docx]

*
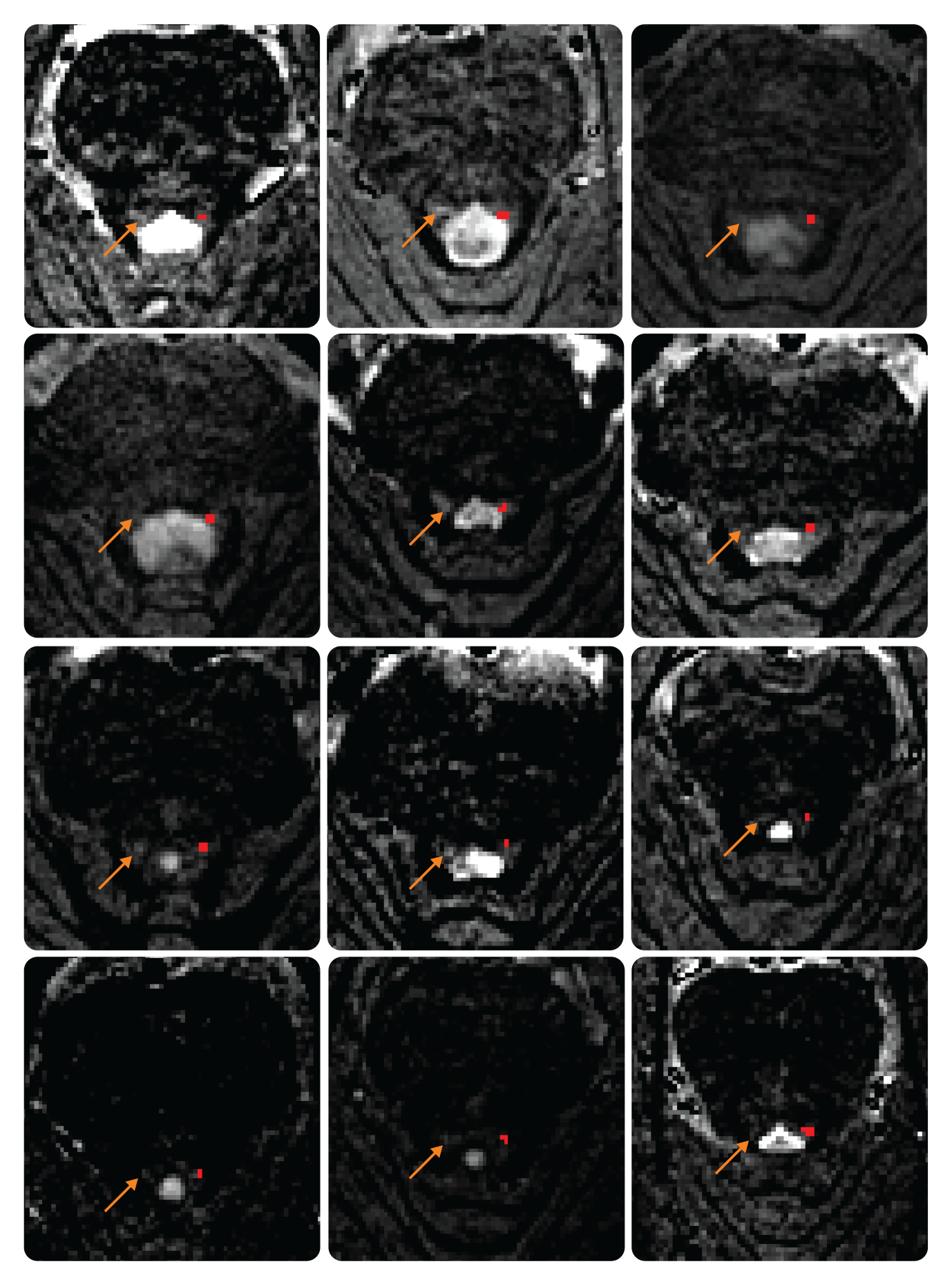
*

**S3 Fig. Depiction of 7T SPIR sequence in native space for each participant separately*.*** *For all participants, an axial slice of the 7T SPIR sequence is shown in native space. The red voxels correspond to the left LC as identified on a 3T TSE scan from scan session 1 and registered to the 7T SPIR volume. The contrast levels were set using the min/max values directly surrounding the LC mask. The arrow indicates the location of the right LC.*
